# CpG-Oligodeoxynucleotides Alleviate Tert-Butyl Hydroperoxide-Induced Macrophage Apoptosis by Regulating Mitochondrial Function and Suppressing ROS Production

**DOI:** 10.1155/2020/1714352

**Published:** 2020-05-09

**Authors:** Yibai Qu, Chunxiu Yang, Xueyang Li, Haihua Luo, Shan Li, Mengwei Niu, Peng Chen, Zhengzheng Yan, Yong Jiang

**Affiliations:** ^1^Guangdong Provincial Key Laboratory of Proteomics, State Key Laboratory of Organ Failure Research, School of Basic Medical Sciences, Southern Medical University, Guangzhou 510515, China; ^2^Department of Anesthesiology, Nanfang Hospital, Southern Medical University, Guangzhou 510515, China

## Abstract

Oxidative stress and mitochondrial dysfunction are related to disease pathogenesis. Oligodeoxynucleotide containing CpG motifs (CpG ODN) demonstrate possibilities for immunotherapy applications. The aim of the present work is to explore the underlying mechanism of the cytoprotective function of CpG ODN by employing the oxidative stress modulation in immune cells. We used the imaging flow cytometry to demonstrate that tert-butyl hydroperoxide (t-BHP) induces mitochondrial-mediated apoptosis and ROS production in RAW264.7 cells. After pretreatment with CpG ODN, the percentage of apoptotic cells and ROS production was both markedly reduced. The decrease in mitochondrial membrane potential (MMP) induced by t-BHP was partially reversed by CpG ODN. The t-BHP induced upregulation of the expression of apoptosis-related proteins (cleaved-caspase 3, cleaved-caspase 9, cleaved-PARP, and bax) was notably decreased in the presence of CpG ODN. Furthermore, we found that CpG ODN enhanced phosphorylation of ERK1/2 and Akt to inhibit ROS production. In conclusion, the protective effect of CpG ODN in mitigation of t-BHP-induced apoptosis is dependent on the reduction of ROS.

## 1. Introduction

Oxidative stress and mitochondrial dysfunction are potentially related to the pathogenesis of various diseases, such as cardiovascular disease, ischemia/reperfusion injury, alcoholic hepatitis, diabetes, Parkinson's disease, and age-related macular degeneration (AMD) [1]. The accumulation of intracellular reactive oxygen species (ROS) may play a key role in the advancement of these diseases.

Reactive oxygen species, which are usually generated by enzymes such as nicotinamide adeninedinucleotide phosphate(NADPH) oxidases, function as both harmful and beneficial species. On one hand, overproduction of ROS is a harmful process that causes injury to cellular components, including DNA, proteins, lipids, mitochondria, and membrane structures. On the other hand, when occurred at moderate concentrations, ROS could play an important role in cellular physiological responses to extracellular stimuli, such as defense against infectious agents [2].

Oxidative stress can also directly activate the inflammatory response and induce mitochondrial (mt) dysfunction. Conversely, mitochondrial dysfunction aggravates oxidative stress and causes the membrane permeability transition (MPT) process and mtDNA translocation to the cytoplasm [3]. Excess ROS generation and release cause a series of oxidative stress responses, which serve to further aggravate mitochondrial dysfunction, leading to cell injury and death [4], subsequently accelerating disease progression [5]. It is well-known that oxidizing agents can induce cell apoptosis, including macrophages and hepatocytes [6].

Macrophage activation in the course of immune and inflammatory responses plays a central role in both innate and adaptive immunity [7, 8]. The functions of activated macrophages include host defense against pathogens, inflammatory reactions, antigen presentation, tissue remodeling, wound healing, and blood lipid homeostasis [9–11]. These immune cells need to perform physiological function under deleterious conditions with ROS production to eliminate pathogen-associated molecular patterns (PAMPs) and/or damage-associated molecular patterns (DAMPs). Simultaneously, macrophages maintain integrity of cell structure and normal biological function. Excessive ROS accumulation causes macrophage dysfunction which leads eventually to cell death; however, macrophages exhibit a sustained survival in this hostile environment via a complex network of protective mechanisms [12].

Oligodeoxynucleotide containing CpG motifs (CpG ODN) were discovered by M. Krieg et al. in 1995. Immunotherapy with CpG ODN demonstrates considerable potential for therapeutic applications. For instance, CpG ODN is used as monotherapy in the prevention of infectious disease and as an adjuvant for allergy, vaccines, and anti-tumor effects in humans [13].

After the discovery of the CpG motif, toll-like receptor (TLR) 9 was identified as the receptor of the immune stimulatory effects of CpG ODN in mouse cell and human cell. In fact, CpG DNA colocalizes with TLR9 in endosomal vesicles [14–16] and activates both host innate and adaptive immune defense mechanisms. In this regard, the main characteristic of the TLR9-induced innate immune response is promotion of the progress of strong-type 1 T helper cell (Th1) adaptive immune responses, including antigen-specific antibodies and CD8^+^ T cell responses [17]. The recognition of CpG motifs requires TLR9, which activates alterations in cellular redox balance and triggers intracellular signaling cascades involved with the mitogen-activated protein kinases (MAPKs) [18].

The immune stimulatory effects of CpG motifs are not a nonspecific toxicity but are essentially highly evolved immune defense mechanisms in support of infection prevention. This includes a broad range of interactive pathways aimed at eliminating pathogenic microorganisms and infectious agents. The beneficial effects of CpG ODN provide the foundation for vaccine improvement and are under investigation in ongoing clinical trials as an immunotherapeutic for infectious diseases, cancer, and allergy. Although CpG ODN is described as potential immunotherapeutic vaccines for many diseases, whether they will provide a protective role in oxidative stress-induced cell apoptosis is still unknown.

Tert-butyl hydroperoxide (t-BHP), an organic peroxide extensively used in many oxidation processes, is considered as a better substitute for hydrogen peroxide (H_2_O_2_) in oxidative stress studies due to its stability [19]. In the present study, we investigated the proapoptotic effect of t-BHP on immune cells and the protective effect of CpG ODN in t-BHP-induced apoptosis and the possible underlying mechanisms. Our study sheds new light on the mechanisms by which CpG ODN act to inhibit t-BHP-induced ROS production and macrophage apoptosis.

## 2. Materials and Methods

### 2.1. Reagents

Cell counting kit-8 (CCK-8) and Annexin V-FITC/PI apoptosis detection kits were purchased from Vazyme Biotechnology Company (Nanjing, China). T-BHP, dimethyl sulfoxide (DMSO), and 2′,7′-dichlorodihydrofluorescein diacetate (DCFHDA) were purchased from Sigma-Aldrich (St. Louis, MO, USA). Cisplatin and N-acetylcysteine (NAC) were purchased from Macklin Biochemical Corporation (Shanghai, China). CpG ODN1826 was purchased from InvivoGen (San Diego, CA, USA). The JC-1 kit was purchased from Beyotime (Shanghai, China). RAW264.7 and AML12 (alpha mouse liver 12) cells were received from American Type Culture Collection (Manassas, VA, USA). Dulbecco's Modified Eagle Medium (DMEM), Dulbecco's phosphate-buffered saline (DPBS), fetal bovine serum (FBS), and Opti-MEM were purchased from Gibco (Waltham, MA, USA). Antibodies for cleaved-caspase 3, caspase 3, caspase 8, cleaved-caspase 8, caspase 9, cleaved-caspase 9, PARP, cleaved PARP, phosphorylated ERK1/2 (p-ERK1/2), ERK1/2, phosphorylated Akt (p-Akt), phosphorylated p38 MAPK, p38 MAPK, phosphorylated JNK, JNK, anti-rabbit IgG, anti-mouse IgG, and GAPDH were all purchased from Cell Signaling Technology (Beverly, MA, USA). Anti-NOX2 antibody (ab80508) was purchased from Abcam (Cambridge, MA, USA). AST and ALT detection kits were purchased from Nanjing Jiancheng Bioengineering Institute (Nanjing, China).

### 2.2. Animal Model

Wild-type (WT) C57BL/6 mice aged 6-8 weeks (20~22 g) were purchased from the experimental animal center of Southern Medical University (Guangzhou, China). All mice were maintained in specific pathogen-free conditions and were housed in a temperature-controlled colony room on a 12/12-hour light-dark cycle. Polymicrobial sepsis was induced by mild cecal ligation and puncture (CLP) as described [20]. Serum and peritoneal macrophage were obtained after mice sacrificed. All animal experiments were approved by the Animal Welfare and Ethics Committee of Southern Medical University, Guangzhou, China.

### 2.3. AST/ALT Measurement

The measurement of AST and ALT was performed according to the user guide of AST and ALT detection kits.

### 2.4. Cell Culture

RAW264.7 and AML12 cells were cultured in DMEM with 10% FBS at 37°C in a humidified incubator under 5% CO_2_. The media were replaced every 2-3 days. All assays were conducted using low-cell passage cells (3-5 passages).

### 2.5. Cell Viability Assay

Cell viability was detected using CCK-8 cell counting kit. Cell suspensions (100 *μ*L/well) were seeded in 96-well plates at a density of 3 × 10^4^ cells/well for cell adherence. After overnight incubation, media were removed from each well and plates treated with t-BHP for three hours or pretreated with CpG ODN for one hour. Ten microliters of CCK-8 solution was added to each well of the plate, and plates were incubated for four hours. Absorbances were read at 450 nm using a SpectraMax M5 microplate reader (Molecular Devices, Waltham, MA, USA).

### 2.6. Apoptosis Assay

Apoptosis was detected by an Annexin V-FITC/PI apoptosis detection kit according to the manufacturer's instructions. Briefly, cells (3 × 10^6^ cells/well) were seeded in 6 cm dishes and pretreated with or without CpG ODN (500 nM), and then subjected to t-BHP (500 *μ*M) stimulation. Cells were collected after incubation with Annexin V-FITC/PI solution for 15 minutes at room temperature. Percentages of apoptotic cells were detected using the Amnis ImageStream Mark II Imaging Flow Cytometer (Luminex Corporation, Waltham, MA, USA).

### 2.7. Intracellular ROS Measurement

RAW264.7 cells were seeded in 6-well plates (3 × 10^5^ cells/well) for cell adherence. After overnight incubation, media were removed and serum-free DMEM was substituted. Cells were then incubated with 10 *μ*M of DCFHDA for 30 minutes and were subsequently treated with t-BHP to induce ROS production. Production of t-BHP-induced ROS was detected by DCFHDA probe using the Amnis ImageStream Mark II Imaging Flow Cytometer, and the fluorescence was observed under a fluorescence microscope (Carl Zeiss, Germany).

### 2.8. Mitochondrial Membrane Potential Assay

A mitochondrial membrane potential (MMP) assay kit with JC-1 was used to detect changes in MMP. Briefly, RAW264.7 cells were seeded in 6 cm dishes (3 × 10^6^ cells/well) for cell adherence overnight. After treatment with t-BHP (500 *μ*M) or CpG (500 nM), cells were incubated with the JC-1 probe for 20 minutes in the cell incubator at 37°C. Fluorescence intensity was monitored using the Amnis ImageStream Mark II Imaging Flow Cytometer. Decreases in red fluorescence and increases in green fluorescence indicated a decrease of MMP.

### 2.9. Western Blotting Assay

RAW264.7 cells were seeded in 6 cm dishes (3 × 10^6^ cells/well) for cell adherence. After overnight incubation, the total protein was extracted by cell lysis buffer after treatment with t-BHP or CpG. Protein in the samples was quantitated by a BCA protein assay kit (Thermo Fisher, Waltham, MA, USA) using a SpectraMax M5 microplate reader [21–23]. Thirty micrograms of protein was subjected to SDS-PAGE and transferred onto PVDF membranes. After blocking with 5% nonfat milk or BSA in Tris-buffered saline tween-20 (TBS-T) at room temperature for 1 h, the membranes were incubated with specific primary antibodies and secondary antibodies of goat anti-rabbit or goat anti-mouse at 4°C overnight. Protein bands were detected by an Immobilon Western horseradish peroxidase (HRP) protein substrate (Merck Millipore, Billerica, CA, USA) and imaged with ChemiDoc™ Touch imaging system (Bio-Rad, Hercules, CA, USA).

### 2.10. Statistical Analysis

The differences between groups were analyzed using Prism 7.0 (GraphPad Software Inc., San Diego, CA), and the statistical analysis was performed by one-way analysis of variance (ANOVA) followed by Tukey's multiple comparisons test. *P* < 0.05 was considered statistically significant. Data were expressed as mean ± SEM of three replicates. Each experiment was repeated at least three times.

## 3. Results

### 3.1. The Cytotoxicity of T-BHP in RAW264.7 Cells

**Figure 1:**
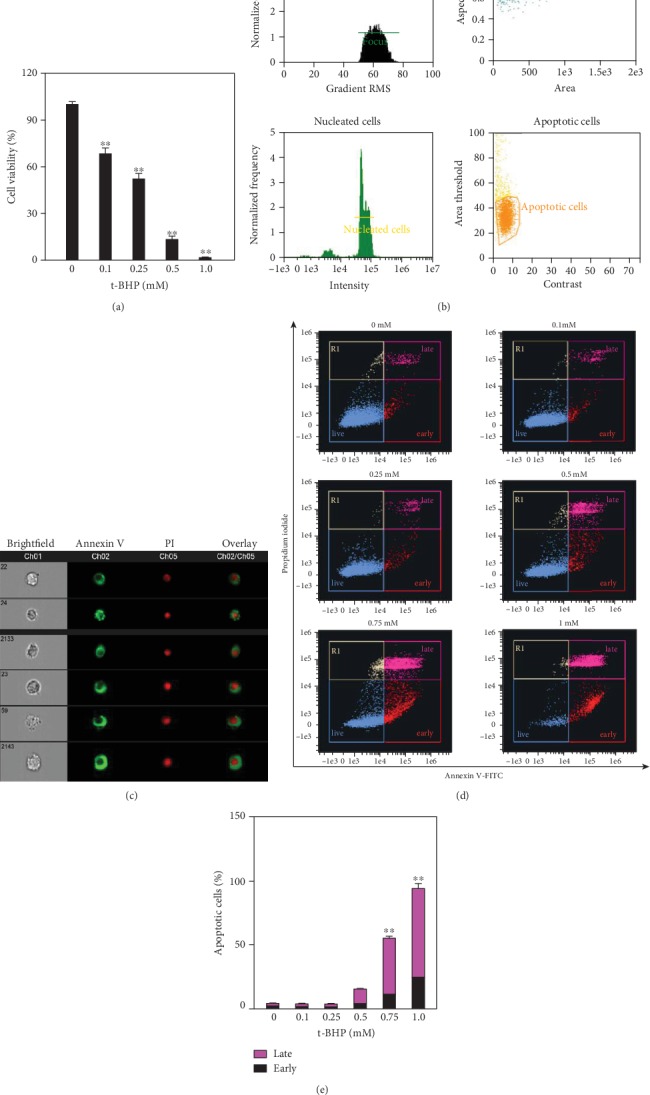
T-BHP induced RAW264.7 cell apoptosis. RAW264.7 cells were exposed to a range of concentrations (0, 0.1, 0.25, 0.5, and 1 mM) of t-BHP for 3 h, the cell viability were assayed by CCK-8 kits (a). After cells were treated with different concentrations (0, 0.1, 0.25, 0.5, 0.75, and 1 mM) of t-BHP for 3 h, and the percentage of cell apoptosis was measured by the Annexin V-FITC/PI apoptosis detection kit and analyzed using the Amnis ImageStream Mark II Imaging Flow Cytometer workflow (b-e). The apoptotic cells were chosen based on gating focused cells, single cells, and nucleated cells step by step (b). The representative image of apoptotic cells derived from imaging flow cytometry was shown at 40x magnification (c).The scatter plot diagrams of apoptosis are analyzed by IDEAS™ v6.2, and the histogram was required from GraphPad prism 7.0 (d, e). The results were presented as mean ± SEM of three independent experiments. ^∗^*P* < 0.05; ^∗∗^*P* < 0.01, *n* = 3.

RAW264.7 cells were exposed to different concentrations of t-BHP for three hours. The CCK-8 assay showed that t-BHP significantly decreased cell viability in a dose-dependent manner (Figure 1(a)). We determined that apoptosis was the primary cell death form in the stimulation of t-BHP using Annexin V and propidium iodide (PI) staining (Figures 1(b) and 1(c)). Figures 1(d) and 1(e) present that the number of apoptotic macrophages was increased with the increase of t-BHP concentration.

### 3.2. T-BHP Activates Apoptosis-Related Caspase Cascades in RAW264.7 Cells

**Figure 2:**
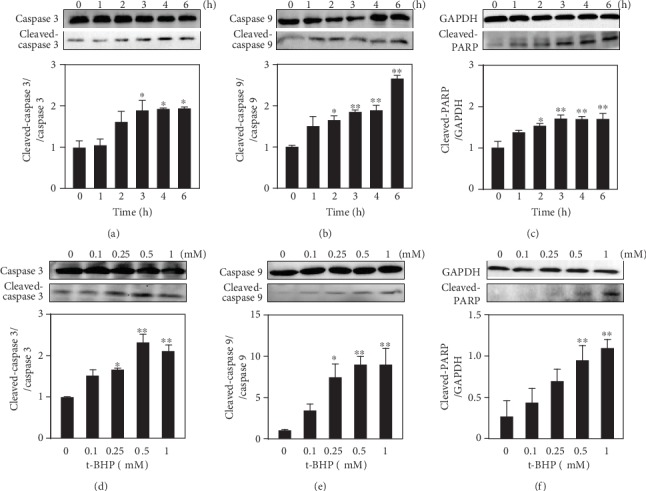
The expression of apoptosis-related proteins induced by t-BHP was detected by Western blotting. RAW264.7 cells were treated with 500 *μ*M t-BHP by time course (0, 1, 2, 3, 4, and 6 h). The expression of cleaved-caspase 9, cleaved-caspase 3, and cleaved-PARP was measured by Western blotting (a–c). Then, cells were treated with different concentrations of t-BHP (0, 0.1, 0.25, 0.5, and 1 *μ*M) for 3 h. The expression of cleaved-caspase 9, cleaved-caspase 3, and cleaved-PARP was measured by Western blotting (d–f). All results were expressed as mean ± SEM of three independent experiments. ^∗^*P* < 0.05; ^∗∗^*P* < 0.01, *n* = 3.

There are two well-characterized apoptotic pathways, the intrinsic and extrinsic pathways. Oxidative stress mainly triggers the intrinsic pathway which is associated with mitochondria-initiated events. Stimuli cause changes in the inner mitochondrial membrane that result in an opening of the MPT pore. The activation of caspase is a critical step in the apoptotic cascade. Therefore, we examined the protein activation levels of caspase 3, caspase 8, caspase 9, and PARP in t-BHP-treated RAW264.7 cells by Western blotting. As illustrated in Figure 2(a), macrophage apoptosis was induced rapidly and occurred within two hours of treatment with t-BHP, and the protein activation level of cleaved-caspase 3 was increased as the exposure time to t-BHP was prolonged. There was no signal of cleaved-caspase 8 detected by Western blotting (data not shown). We subsequently investigated caspase 9 activation at different time points of t-BHP treatment, and the results confirmed the involvement of caspase 9 in t-BHP-induced apoptosis (Figure 2(b)); PARP is a 116 kDa protein in its intact form and can be cleaved to an 85 kDa fragment and induced by the cleavage of caspase 3 [24]. We also determined the activation of PARP (Figure 2(c)). We observed that the degree of caspase 9 and PARP activation was also time-dependent upon 500 *μ*M t-BHP treatment. We also measured the activation levels of caspase proteins after three hours of treatment with different concentrations of t-BHP, and the results showed that the activation level of caspase protein also increased gradually over time with increases in treatment concentrations (Figures 2(d)-2(f)).

### 3.3. The T-BHP Induced Dose-Dependent Production of Intracellular ROS

**Figure 3:**
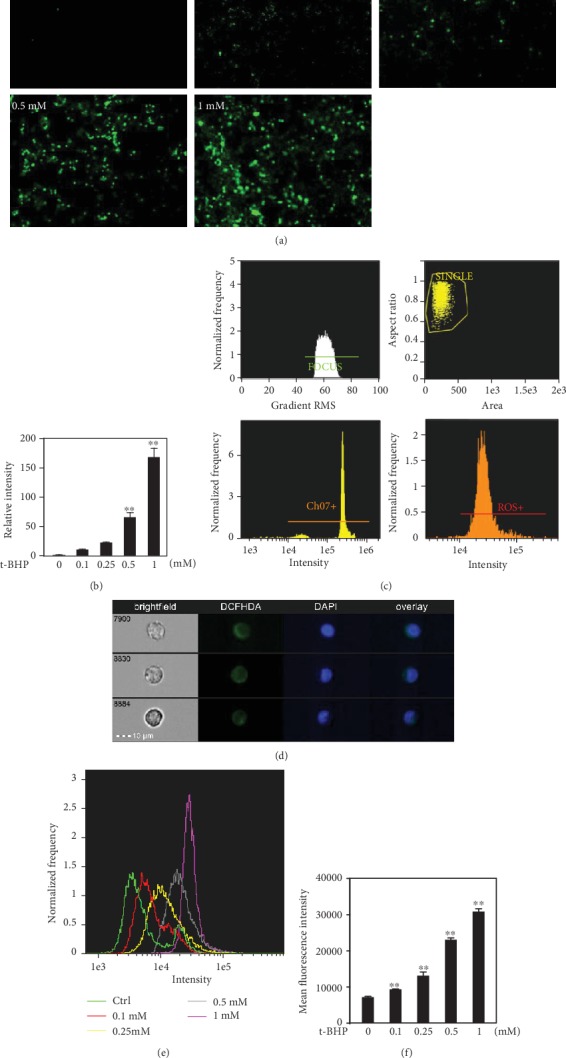
The production of ROS was required for t-BHP-induced apoptosis. RAW264.7 cells were treated with a range of t-BHP (0, 0.1, 0.25, 0.5, and 1 mM) for 1 h. ROS release was detected by DCFHDA probe using a fluorescence microscope at 100x magnification (a, b) and imaging flow cytometry at 40x magnification (c–f). All results were expressed as mean ± SEM of three independent experiments. ^∗^*P* < 0.05; ^∗∗^*P* < 0.01, *n* = 3.

The release of ROS is a critical event in oxidant-induced cytotoxicity [2]. Therefore, the role of cellular ROS in t-BHP-induced macrophage apoptosis was further investigated. Treatment of t-BHP to the macrophages resulted in a significant increase in free radical production as measured in terms of fluorescence of the DCFHDA probe, and the production of ROS was generally increased in a dose-dependent manner as shown in Figures 3(a) and 3(b). ROS production was also analyzed by imaging flow cytometry; the results showed that significant increases in DCFHDA fluorescence indicated increases of intracellular ROS in RAW264.7 cells exposed to a high dose of t-BHP (Figures 3(c)-3(f)). In addition, to determine whether cell apoptosis was induced by ROS after treatment with t-BHP, NAC was used for apoptosis assay as a ROS inhibitor. After treatment with NAC, the percentage of apoptotic cells was decreased dramatically (Supplementary Figure S1). It demonstrated that the cell apoptosis was induced by excess ROS production.

### 3.4. CpG ODN Inhibited T-BHP-Induced Cell Apoptosis and Reversed Mitochondrial Dysfunction in RAW264.7 Cells

**Figure 4:**
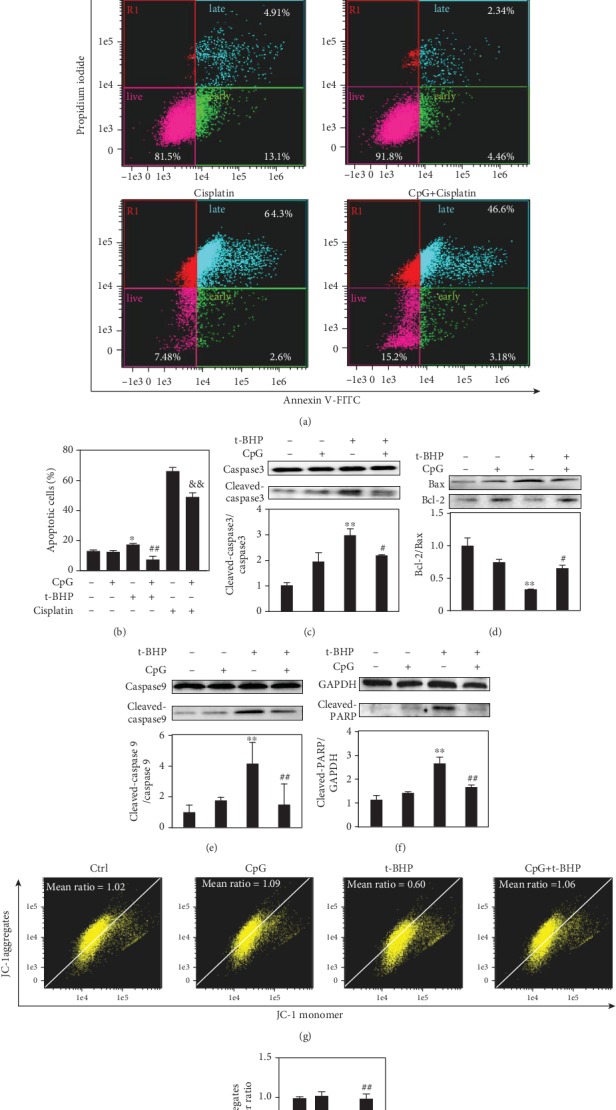
CpG ODN decreased cell apoptosis and reduced apoptosis-related protein expression. Cisplatin-induced apoptosis was used as positive control. RAW264.7 cells were pretreated with CpG ODN (500 nM) for 1 h, after stimulation of t-BHP (500 *μ*M) for 6 h and cisplatin (40 *μ*g/ml) for 24 h. The percent of apoptotic cells was measured by the Annexin V/PI dye and analyzed by the Amnis ImageStream Mark II Imaging Flow Cytometer workflow. The scatter plot diagrams of apoptosis are analyzed by IDEAS™ v6.2, and the histogram was required from GraphPad prism 7.0 (a, b). Apoptosis-related protein expression (bax, bcl-2, cleaved-caspase 9, cleaved-caspase 3, and cleaved PARP) was measured by Western blotting (c-f). Change of mitochondrial membrane potential was assayed by JC-1(g, h). All results were expressed as mean ± SEM of three independent experiments. ^∗^*P* < 0.05; ^∗∗^*P* < 0.01, versus the control group. ^#^*P* < 0.05; ^##^*P* < 0.01, versus the t-BHP group. ^&&^*P* < 0.01, versus the cisplatin group. *n* = 3.

Based on previous studies of CpG ODN in protection against infectious disease, we explored whether it plays an important role in cell apoptosis induced by oxidative stress to investigate the potential cytoprotective effect of CpG ODN. The image flow cytometry results showed that pretreatment with 500 nM CpG ODN for one hour could protect the cells against t-BHP-induced apoptosis. Meanwhile, the cisplatin-induced apoptosis was used as positive control in apoptosis assay (Figures 4(a) and 4(b)). The percentage of apoptotic cells induced by cisplatin was also dramatically decreased after treatment with CpG ODN in RAW264.7 macrophages. Moreover, we tested the expression of related apoptotic proteins to explore the possible functions of CpG ODN in antiapoptosis effects. Results showed that CpG ODN dramatically decreased bax protein expression and decreased the formation of cleaved-caspase 3, cleaved-caspase 9, and cleaved-PARP, whereas bcl-2 protein expression was increased (Figures 4(c)–4(f)). Mitochondrial dysfunction was involved in t-BHP-linked cell death. We next detected the change in MMP using JC-1 dye, and t-BHP-induced MMP were observed dramatically decreased, while CpG ODN inhibited the loss of MMP (Figures 4(g) and 4(h)). In addition, we explored whether CpG ODN performed the same cytoprotective effect in other cell lines. Then, the AML12 cell line, which is a murine hepatocyte cell line, was used for apoptosis assay. We detected the cell viability in treatment with different concentrations (0, 0.2 mM, and 0.5 mM) of t-BHP by CCK-8 assay (Supplementary Figure S2(a)). Results showed that cell viability was decreased with the increase of t-BHP concentration. We also performed the cell apoptosis assay experiment by imaging flow cytometry (Supplementary Figures S2(b) and 2(c)). The result showed that the role of CpG ODN in t-BHP-induced apoptosis of the AML12 cell line was opposite compared with that in macrophages. It indicated that the cytoprotective effect of CpG ODN may differ in different cell types. The detailed mechanism need to be further explored.

### 3.5. Phosphorylation of ERK1/2 and Akt Was Involved in T-BHP-Induced RAW264.7 Cell Apoptosis

Mitogen-activated protein kinases (MAPKs), including p38 MAPK, extracellular signal-regulated kinases (ERKs), and c-Jun N-terminal kinases (JNKs), are sensitive to oxidative stress and play key roles in cell survival, proliferation, and death [25]. Activation of Akt is associated with cell apoptosis and survival [2].

**Figure 5:**
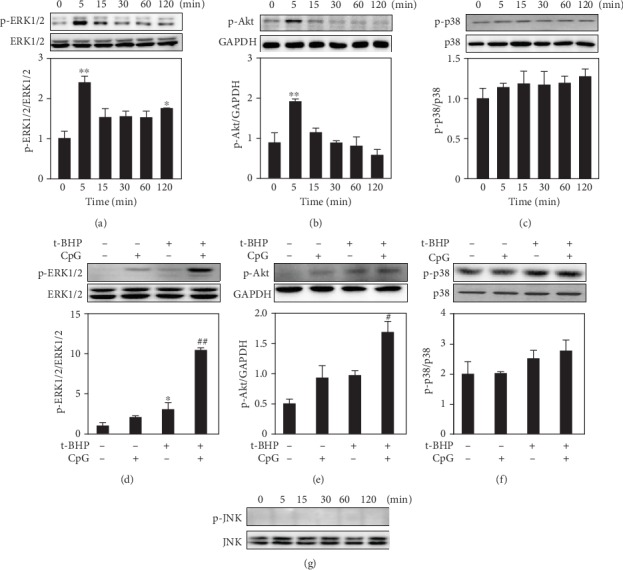
The activation of ERK1/2 and Akt was involved in t-BHP induced RAW264.7 cell apoptosis. The effect of t-BHP on activating MAPKs signaling pathways was determined by Western blotting in time course (a-c). Synergistic effects on the phosphorylation level of ERK1/2 and Akt were observed by Western blotting (d-f). All results were expressed as mean ± SEM of three independent experiments. All results were expressed as mean ± SEM of three independent experiments. ^∗^*P* < 0.05; ^∗∗^*P* < 0.01, *n* = 3.

Thus, in order to verify the key molecules involved in CpG ODN effects against t-BHP-induced apoptosis, the changes in phosphorylation levels of MAPKs and Akt were determined by Western blotting analysis. As shown in Figures 5(a)-5(c), when RAW264.7 cells were exposed to 500 *μ*M t-BHP at different time points, the phosphorylation levels of ERK1/2 and Akt were increased at five minutes as compared to those of other groups. Alteration of the phosphorylation level of p38 MAPK was not significant between each group. Moreover, our data showed that CpG ODN was able to significantly increase the phosphorylation level of ERK1/2 and Akt (Figures 5(d)-5(f)). However, the phosphorylation of JNK could not be detected in all groups (Figure 5(g)).

### 3.6. CpG ODN Protects RAW264.7 Cells against T-BHP-Induced Apoptosis by Increasing the Phosphorylation of ERK1/2 and Akt

**Figure 6:**
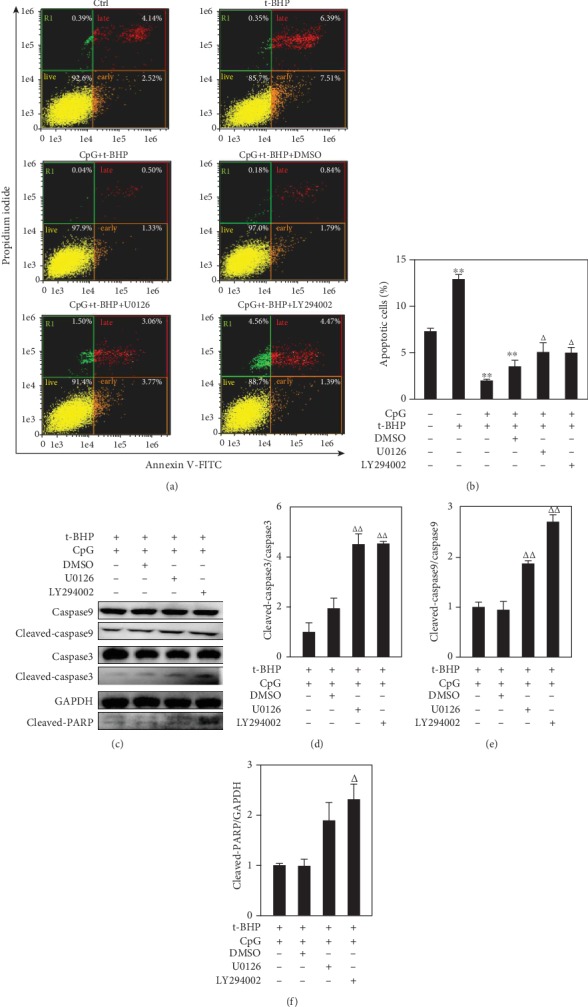
Changes of the phosphorylation of ERK1/2 and Akt played dual roles in response to t-BHP-induced apoptosis. Apoptotic cells were detected by imaging flow cytometry (a, b), and apoptotic-related proteins were assayed by Western blotting (c-f) in the use of the inhibitors of ERK1/2 (U0126, 10 *μ*M) and Akt (LY294002, 10 *μ*M). All results were expressed as mean ± SEM of three independent experiments. ^∗^*P* < 0.05; ^∗∗^*P* < 0.01, versus the control group. ^#^*P* < 0.05, ^##^*P* < 0.01, versus the t-BHP group. *^Δ^P* < 0.05, *^ΔΔ^P* < 0.01, versus the t-BHP+CpG group. *n* = 3.

To further investigate the antiapoptotic mechanism of CpG ODN, inhibition of phosphorylation was performed by using specific inhibitors against ERK1/2 and Akt signaling pathways, U0126, and LY294002, respectively. Results showed that U0126 and LY294002 both have significant inhibitory effects on t-BHP-induced RAW264.7 cell apoptosis (Figures 6(a) and 6(b)). Analysis of apoptotic protein expression shows that the production of cleaved-caspase 9, cleaved-caspase 3, and cleaved-PARP was markedly increased after using inhibitors of ERK1/2 and Akt (Figures 6(c)-6(f)).

### 3.7. The Protective Effect of CpG ODN from T-BHP-Induced Oxidative Damage Is Mediated by the Activation of ERK1/2 and Akt

**Figure 7:**
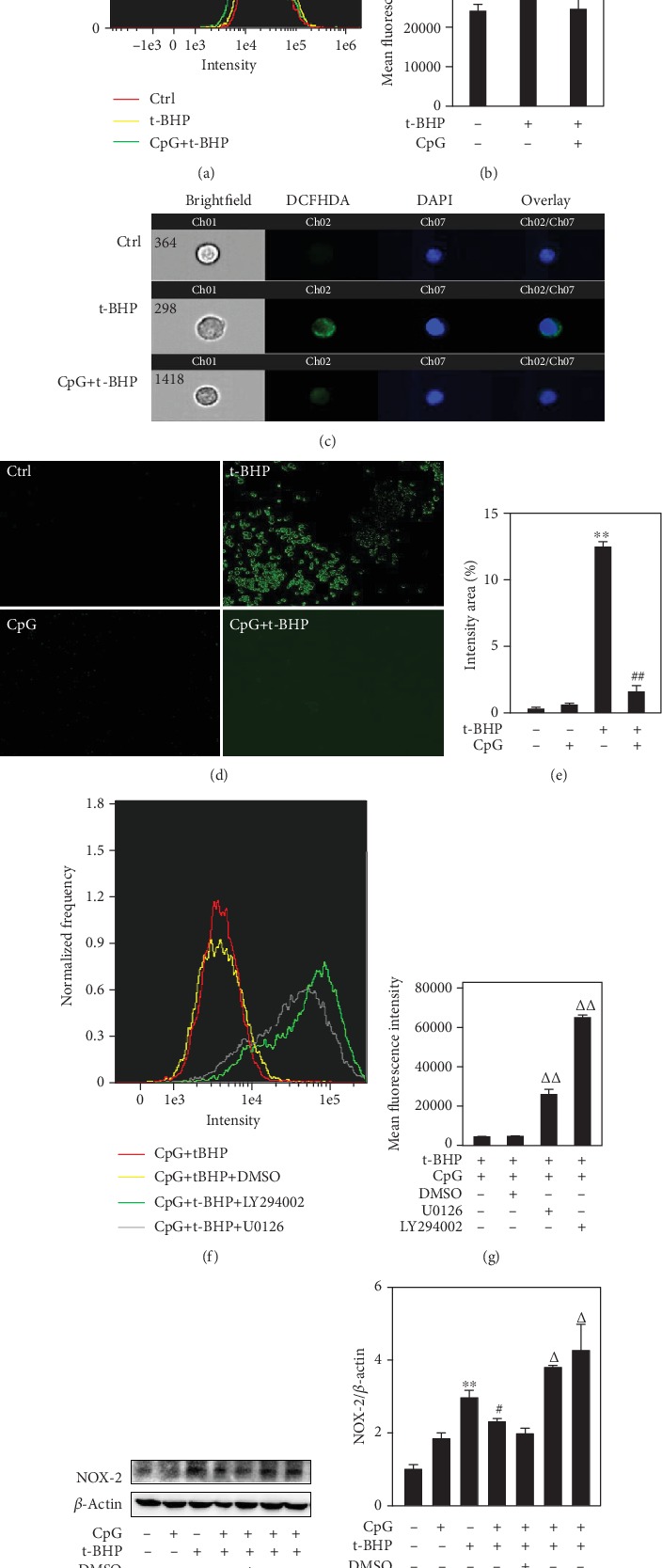
The protective effect of CpG ODN in response to t-BHP-induced oxidative damage. RAW264.7 cells were pretreated with CpG ODN (500 nM) for 1 h and then exposed to t-BHP (500 *μ*M) for 1 h. ROS production was measured by imaging flow cytometry at 40x magnification (a-c) and fluorescence microscope at 100x magnification (d, e). ROS was also measured in the use of the inhibitors of ERK1/2 (U0126, 10 *μ*M) and Akt (LY294002, 10 *μ*M) (f, g). Then, NOX2 expression was determined in the use of the inhibitors of ERK1/2 (U0126, 10 *μ*M) and Akt (LY294002, 10 *μ*M) by Western blotting after treatment with or without CpG and t-BHP for one hour, respectively (h, i). All results were expressed as mean ± SEM of three independent experiments. ^∗^*P* < 0.05; ^∗∗^*P* < 0.01 versus the control group. ^#^*P* < 0.05, ^##^*P* < 0.01, versus the t-BHP group. *^Δ^P* < 0.05, *^ΔΔ^P* < 0.01 versus the t-BHP+CpG group. *n* = 3.

We then sought to determine whether the inhibitory effect of CpG ODN on intracellular ROS generation was mediated by p-ERK1/2 and p-Akt. According to flow cytometry images, t-BHP-induced ROS production was markedly decreased by CpG ODN as compared to the t-BHP group (Figures 7(a)-7(c)). Similar results were observed using the fluorescence microscope (Figures 7(d) and 7(e)). In addition, we used the inhibitors of ERK1/2 and Akt to observe the inhibitory effect on intracellular ROS generation. Both inhibitors were capable of increasing ROS formation (Figures 7(f) and 7(g)). Our study revealed that ROS generation is reduced by CpG ODN through phosphorylation of ERK1/2 and Akt. Next, we wanted to determine which molecular mechanism is involved with the upstream effectors of ROS generation. In general, the NOX family of NADPH oxidase is an important source of ROS generation. NOX2 was first described in neutrophils and macrophages and is the most widely distributed among the NOX isoforms [26]. Thus, we focused on the changes in NOX2 protein expression using specific inhibitors of ERK1/2 (U0126) and Akt (LY294002). Western blotting results showed that activation of ERK1/2 and Akt inhibits NOX2 protein expression (Figures 7(h) and 7(i)).

### 3.8. CpG ODN Suppress ROS Production Both in Peritoneal Macrophages and Liver of Septic Mice

**Figure 8:**
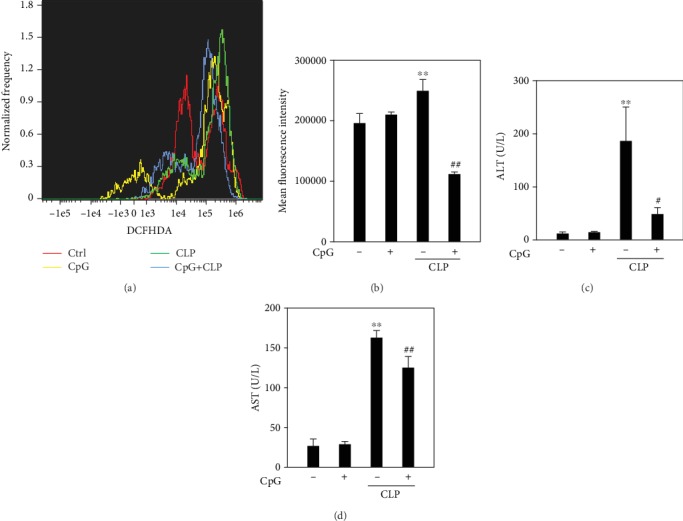
The protective effect of CpG ODN in CLP mice. Mice were pretreated with CpG ODN (30 *μ*g/mice) for one hour by tail vein injection; then, the CLP model was performed. After 12 h, mice were sacrificed and peritoneal macrophages were obtained from peritoneal lavage fluid. ROS production was detected by DCFHDA probe (a, b). Meanwhile, serum was obtained for AST and ALT detection (c, d). ^∗^*P* < 0.05; ^∗∗^*P* < 0.01, versus the control group. ^#^*P* < 0.05, ^##^*P* < 0.01 versus the t-BHP group. *n* = 5.

CpG ODN has been shown to prevent mortality from CLP sepsis [27]. In the livers of the animal model of sepsis, ROS accumulation is involved in the initiation of the mitochondrial apoptotic pathway and liver damage [28]. Then, ROS production in peritoneal macrophages was detected. Results presented that pretreatment of CpG ODN for one hour could reduce ROS accumulation of peritoneal macrophages in CLP mice (Figures 8(a) and 8(b)). In addition, the results of AST and ALT detection imply that CpG ODN can alleviate the septic liver damage (Figures 8(c) and 8(d)).

## 4. Conclusions and Discussion

**Figure 9:**
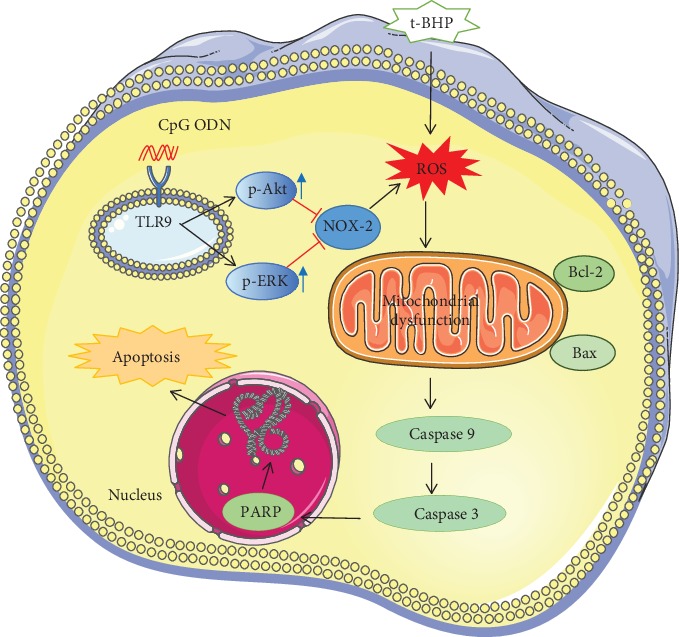
A schematic diagram of the mechanism of CpG ODN in cytoprotective effects.

In our study, the effects of CpG ODN in reducing t-BHP-induced RAW264.7 cell apoptosis were investigated. A schematic diagram of CpG ODN on t-BHP induced macrophage apoptosis is shown in Figure 9. Our major conclusions include (1) t-BHP-induced cell apoptosis and ROS production were dependent on concentration, (2) t-BHP induces apoptosis via the mitochondrial-mediated apoptosis signaling pathway, (3) the protective effect of CpG ODN in reduction of t-BHP-induced apoptosis results from inhibition of ROS production, and (4) changes in the activation of ERK1/2 and Akt were involved in the protective mechanism of CpG ODN. Oxidative stress is a biochemical imbalance between the formation of free radicals and their elimination by endogenous antioxidant defense systems, and it is also a cascade reaction characterized by a significant increase in the amount of oxidized components and plays a basic role in the pathogenesis of various human diseases [29]. Free radicals affect both the structure and function of cells such as epithelial cells, neural cells, and immune cells and may contribute to disease progression of cardiovascular diseases, diabetes, Parkinson's disease, Alzheimer's disease, and others [30–33]. Free radicals or ROS play a vital role in immune responses, gene transcription, and programmed cell death (apoptosis) under normal physiological conditions [34]. Small amounts of ROS do not cause damage but coordinate with the antioxidant system to maintain homeostasis; however, overproduction of ROS results in damage to cell membranes, DNA, proteins, and cellular structures. Release of ROS appears to be a critical event in oxidant-induced cell apoptosis [2]. Likewise, mitochondria at the center of cellular metabolism and as major regulators of redox balance in eukaryotic cells play a critical role in disease development and progression. Oxidative stress-induced damage of mitochondria mediates and accelerates the process of cell death, and subsequently, mitochondrial dysfunction generates excess mitochondrial ROS that causes severe cellular damage and death [5]. Although many pharmacological agents currently in use modulate oxidative stress and improve disease progression by reducing ROS generation and improving antioxidant mechanisms, clinical effects need further development. Hence, exploration of new ways to inhibit oxidative stress may play essential roles in suppressing mitochondrial dysfunction-induced apoptosis and cell damage. There are two general strategies to defend against infectious diseases in the mammalian immune system. Innate immune responses are activated by PAMPs which are expressed by a diverse group of pathogenic microorganisms [35]. TLRs, as a part of the innate immune system, are key in the recognition of PAMPs and initiating sufficient immune responses [36, 37]. Unmethylated CpG motifs, which are mostly presented in microbial DNA, are one of the factors involved in immune responses. Among the TLRs, TLR9 is the major participant in unmethylated CpG oligonucleotide recognition, and CpG ODN-induced TLR9 activation regulates immune cells and exerts anti-inflammatory effects [38–41].

Growing evidence shows that the beneficial effects of CpG ODN provide the foundation for improved vaccine production as well as its use as an immunotherapeutic for infectious diseases, allergies, and cancer, which are under investigation in clinical trials [13]. Several unmethylated CpG motifs are presented in bacterial DNA, which are a type of PAMPs recognized by the innate immune system [42]. Unmethylated CpG motifs were identified as crucial mediators of immune activation, and the study showed that B cells responded to CpG motifs by proliferation and secretion of immunoglobulins [43]. Furthermore, differences in methylation patterns and the use of CpG dinucleotides result in unmethylated CpG motifs being present at a much higher frequency in the genomes of prokaryotes than in the genomes of eukaryotes [44, 45]. These unmethylated CpG motifs were detected by using TLR9 to regulate response of immune cells. Previous studies have demonstrated that TLR9 mediated CpG recognition in mice and in humans [15, 16]. More specifically, CpG ODNs are rapidly internalized by immune cells and are involved with phosphatidylinositol-3-kinases and interact with TLR9 present in endocytic vesicles [15, 46]. The recognition of TLR9 by CpG ODN leads to the swelling and acidification of the endocytic vesicles and generation of ROS. This series of events are critical to CpG-mediated signaling, as agents that inhibit endosomal maturation or acidification (e.g., chloroquine and wortmannin) block immune system activation [47, 48]. Synthetic unmethylated oligodeoxynucleotides that contain CpG motifs which are similar to those discovered in bacterial genomes DNA induce a similar immune response [43, 49, 50]. The immunomodulatory CpG ODN has various potential therapeutic uses in allergy, cancer, and infectious diseases [51].

As a DAMP molecular, CpG ODN has been proved to promote the expression of inflammation related genes and enhance the release of inflammatory cytokines. Plenty studies reported that CpG ODN promotes apoptosis in many cell types, including bladder cancer cells, B cells and lymphoma cells [52–54]. While in some conditions, CpG ODN has been evidenced to inhibit cell apoptosis induced by different stimuli, such as irradiation, starvation and ischemia/reperfusion [55–57]. These studies indicated that CpG ODN plays dual function roles on cell apoptosis which depends on different cell types and conditions. In our study, we demonstrated that CpG ODN promotes the apoptosis of murine hepatocyte AML12 cell under oxidative stress but decreases the apoptosis of macrophage induced by t-BHP. It was reported that CpG ODN induces early hepatic injury but provides a late window for protection against endotoxin-mediated liver damage [58]. According to these studies, we speculate that CpG ODN promotes the apoptosis of hepatocyte during liver injury in early stage, resulting in the release of DAMPs, such as cellular DNA and HMGB1. During a later period, CpG ODN mitigates the apoptosis of macrophage to engulf DAMP molecules, ultimately to protect liver from injury at a late stage. This may be one of the reasonable explanations why CpG ODN plays opposite effects between hepatocytes and macrophages.

Neutrophils play a critical role in the innate immune response to bacterial infections by mediating the phagocytosis and destruction of microorganisms, as well as producing cytokines, chemokines, and growth factors that activate the immune response. Previous studies have shown that CpG ODN inhibits neutrophils migration *in vitro* and leucocyte migration *in vivo* [59]. CpG ODN with different types and structures performed different responses to neutrophils and other cell lines. In a previous study, it was demonstrated that CpG ODN delays apoptosis of neutrophil granulocytes [60]. Studies have also demonstrated the impact of phosphorothioate modification of ODNs on adhesive properties of neutrophils, which unmasks the specific effects of CpG motifs in the structures [61]. However, more exploration on the mechanism of cytoprotective of CpG ODN in human immune cell is required.

In our study, although we have not focused the CpG ODN pharmacokinetics and biodistribution *in vivo*, the results of the animal model in our study have implicated that the liver function injury was partially alleviated by CpG ODN, which further strengthened the effect of CpG ODN *in vivo*. Because of the immune stimulation effect elicited by CpG motifs, the safety issues of CpG ODN in therapy application need to be a concern. Several studies have shown that there are no reports of toxic shock when CpG ODN is administered at concentrations typically present in vaccines [62, 63]. Evidence from clinical trials indicates that CpG ODNs are reasonably safe when administered as vaccine adjuvants. This includes reports showing that conventional and CpG-adjuvanted vaccines have similar safety profiles [64, 65]. Clinical studies have been operated to evaluate the safety and activity of CpG ODNs in humans. Available results suggest that these agents are reasonably safe and could improve the response to prophylactic pathogen-specific vaccines.

As known, t-BHP exposure not only results in cell death by promotion of apoptosis but also leads to oxidative stress by increased ROS production. Our results demonstrate that the cell viability of RAW264.7 cells was reduced after treatment with t-BHP, and cell apoptosis was promoted as compared with the control group, whereas CpG ODN pretreatment remarkably enhanced cell viability and decreased t-BHP-induced cell apoptosis. We observed that CpG ODN significantly decreased ROS production. After using different inhibitors of MAPKs and Akt, we declared that the mechanism for the cytoprotective effect of CpG ODN was through reducing ROS production, which was dependent on the activation of ERK1/2 and Akt, and NOX2 expression inhibition.

In conclusion, through ERK1/2 and Akt signaling pathway, CpG ODN could reduce oxidative stress and cell apoptosis induced by t-BHP in macrophages, indicating CpG ODN may activate the innate immune system as defense against oxidative stress damage in macrophages. For better understanding of the role of CpG ODN in the innate immune system, future studies are needed.

## Data Availability

The data used to support the findings of this study are available from the corresponding author upon request.

## Supplementary Materials

Supplementary Materials Figure 1: cell apoptosis induced by ROS. RAW264.7 cells were pretreated by NAC (5 mM) for 30 min; after stimulation of t-BHP (500 *μ*M) for 1 hour, ROS was measured by DCFHDA probe (1(a) and 1(b)). Meanwhile, apoptosis assay was determined after treating with NAC (5 mM) or (and) t-BHP (500 *μ*M) (1(c) and 1(d)). Apoptosis-related protein cleaved-caspase 3 was detected by Western blot after using NAC (1(e) and 1(f)). All results were expressed as mean ± SEM of three independent experiments. ^∗^*P* < 0.05; ^∗∗^*P* < 0.01. *n* = 3. Supplementary Materials Figure 2: the role of CpG ODN in t-BHP-induced apoptosis of the AML12 cell line. Cisplatin-induced apoptosis was used as positive control. The cell viability of AML12 cell was measured by the CCK-8 kit with different concentrations (0 mM, 0.2 mM, and 0.5 mM) of t-BHP (Supplemental Figure 2(a)). AML-12 cells were pretreated with CpG for 1 hour, after stimulation of t-BHP (500 *μ*M) for 6 hours and cisplatin (40 *μ*g/ml) for 24 hours. The percent of apoptotic cell was measured by the Annexin V/PI dye and analyzed by the Amnis ImageStream Mark II Imaging Flow Cytometer workflow (Supplemental Figures 2(b) and 2(c)). The scatter plot diagrams of apoptosis are analyzed by IDEASTM v6.2, and the histogram was required from GraphPad prism 7.0. All results were expressed as mean ± SEM of three independent experiments. ^∗^*P* < 0.05; ^∗∗^*P* < 0.01; ns = no significant difference, *n* = 3.
