# Supplementary material for: CpG-Oligodeoxynucleotides Alleviate Tert-Butyl Hydroperoxide-Induced Macrophage Apoptosis by Regulating Mitochondrial Function and Suppressing ROS Production
Source: Oxid Med Cell Longev. 2020 May 9;2020:1714352. doi: 10.1155/2020/1714352 (PMC7232733; doi:10.1155/2020/1714352)
Supplement: Supplementary Materials — Figure 1: cell apoptosis induced by ROS. RAW264.7 cells were pretreated by NAC (5 mM) for 30 min; after stimulation of t-BHP (500 μM) for 1 hour, ROS was measured by DCFHDA probe (1(a) and 1(b)). Meanwhile, apoptosis assay was determined after treating with NAC (5 mM) or (and) t-BHP (500 μM) (1(c) and 1(d)). Apoptosis-related protein cleaved-caspase 3 was detected by Western blot after using NAC (1(e) and 1(f)). All results were expressed as mean ± SEM of three independent experiments. ∗P < 0.05; ∗∗P < 0.01. n = 3. Supplementary Materials Figure 2: the role of CpG ODN in t-BHP-induced apoptosis of the AML12 cell line. Cisplatin-induced apoptosis was used as positive control. The cell viability of AML12 cell was measured by the CCK-8 kit with different concentrations (0 mM, 0.2 mM, and 0.5 mM) of t-BHP (Supplemental Figure 2(a)). AML-12 cells were pretreated with CpG for 1 hour, after stimulation of t-BHP (500 μM) for 6 hours and cisplatin (40 μg/ml) for 24 hours. The percent of apoptotic cell was measured by the Annexin V/PI dye and analyzed by the Amnis ImageStream Mark II Imaging Flow Cytometer workflow (Supplemental Figures 2(b) and 2(c)). The scatter plot diagrams of apoptosis are analyzed by IDEASTM v6.2, and the histogram was required from GraphPad prism 7.0. All results were expressed as mean ± SEM of three independent experiments. ∗P < 0.05; ∗∗P < 0.01; ns = no significant difference, n = 3. [file 1714352.f1.doc]

0

2000

4000

6000

******

******

+

t-BHP

**–**

**–**

+

+

NAC

**–**

**–**

+

Mean Intensity

(b)

Supplemental figure 1

(a)


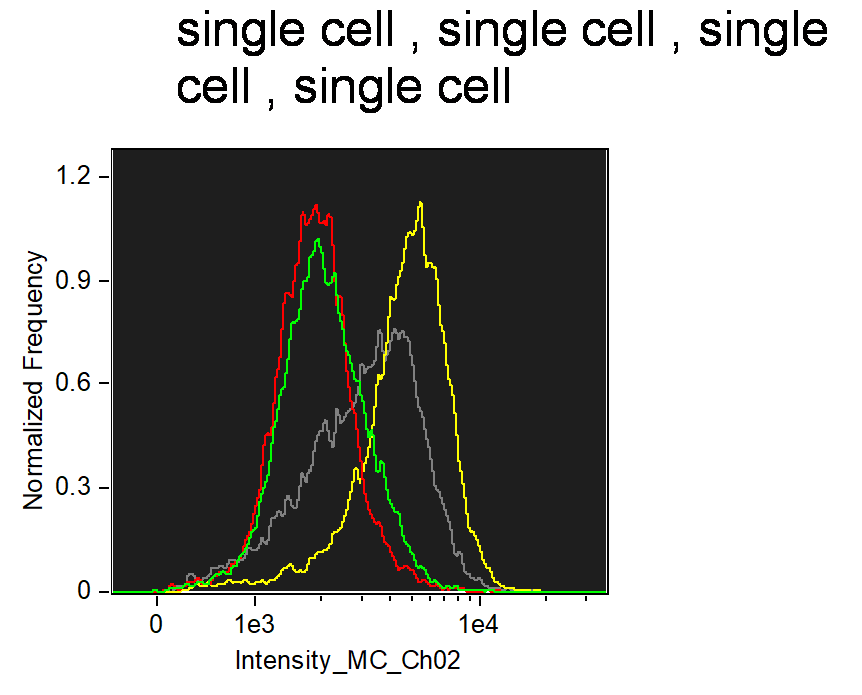


control

t-BHP

NAC

NAC+t-BHP

(d)

Apoptotic cell (%)

0

5

10

15

**

**

+

**–**

**–**

+

+

NAC

**–**

**–**

+

t-BHP

Annexin V-FITC


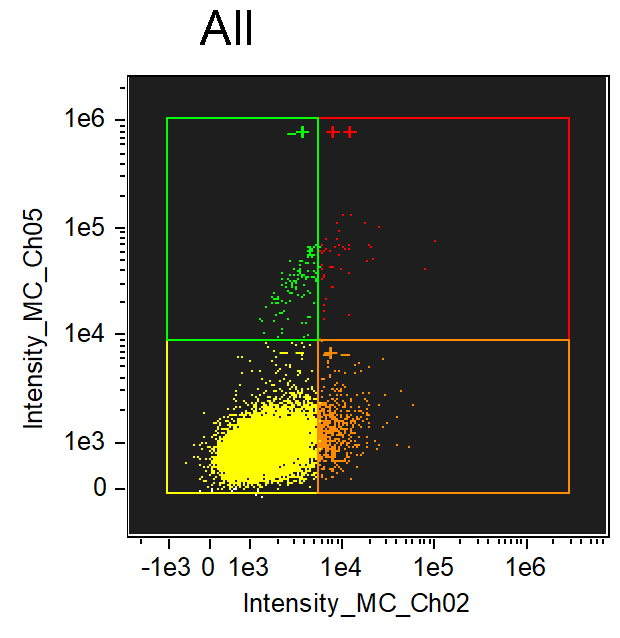

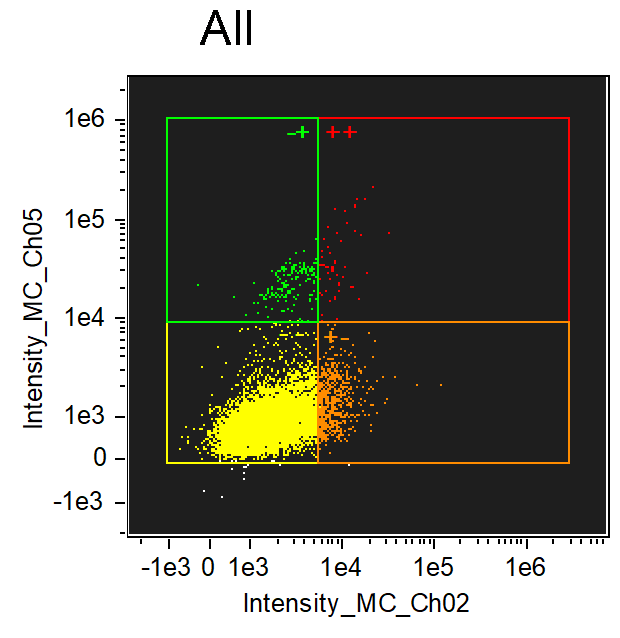

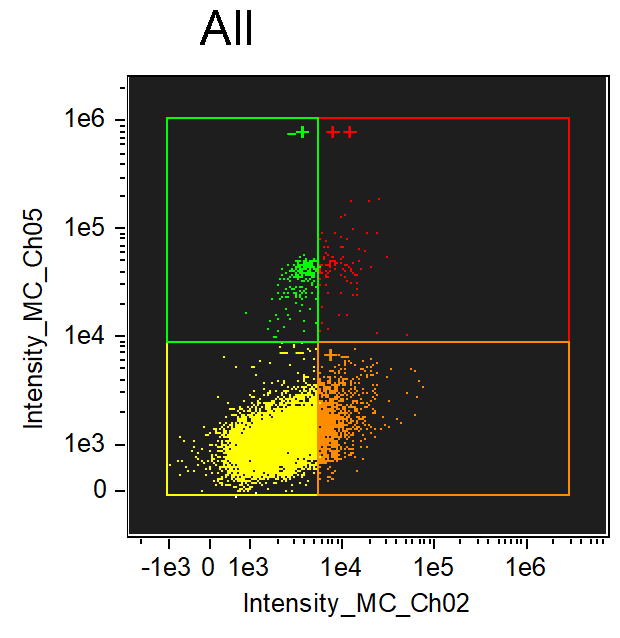

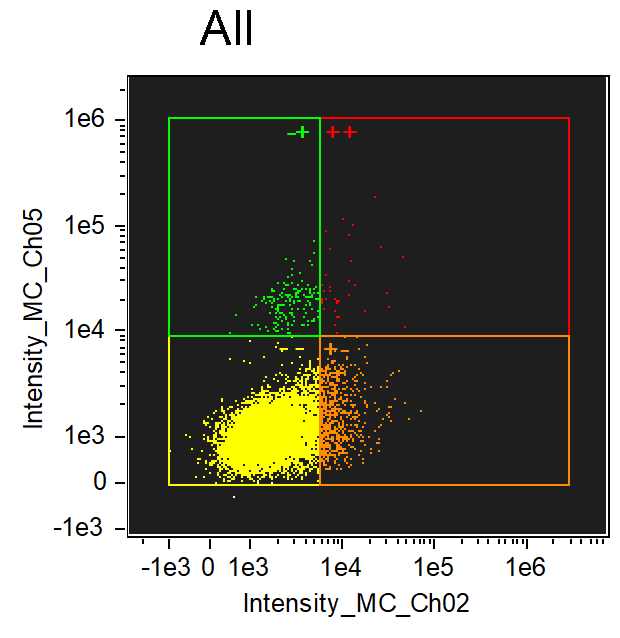


93%

5.25%

0.45%

92.1%

5.51%

0.53%

91%

86.5%

10.5%

0.95%

0.36%

6.87%

t-BHP

NAC+t-BHP

NAC

ctrl

Propidium Iodide

(c)

3

*****

(e)

(f)

Cleaved

caspase3/caspase3

+

**–**

**–**

+

+

NAC

**–**

**–**

+

t-BHP

2


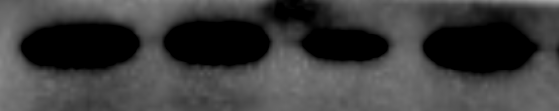


Caspase3


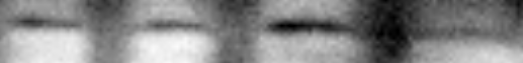


Cleaved

caspase3

1

0

+

**–**

**–**

+

t-BHP

+

NAC

**–**

**–**

+

Supplemental figure 1. Cell apoptosis induced by ROS. RAW264.7 cells were pretreated by NAC (5 mM) for 30 min, after stimulation of t-BHP (500 µM) for 1 hour, ROS was measured by DCFHDA probe (1(a) and 1(b)). Meanwhile, apoptosis assay were determined after treating with NAC (5 mM) or (and) t-BHP (500 µM) (1(c)-1(d)). Apoptosis related protein cleaved caspase 3 was detected by western blot after using NAC (1(e) and 1(f)). All results were expressed as mean ± SEM of three independent experiments. ^*^ *P* < 0.05; ^**^ *P* < 0.01. n = 3

Supplemental figure 2

t-BHP


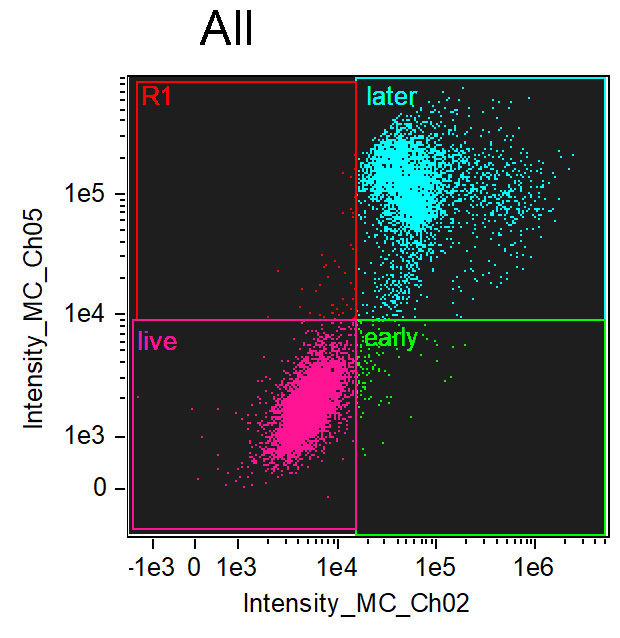


41.7%

1.19%

56.7%

CpG+t-BHP


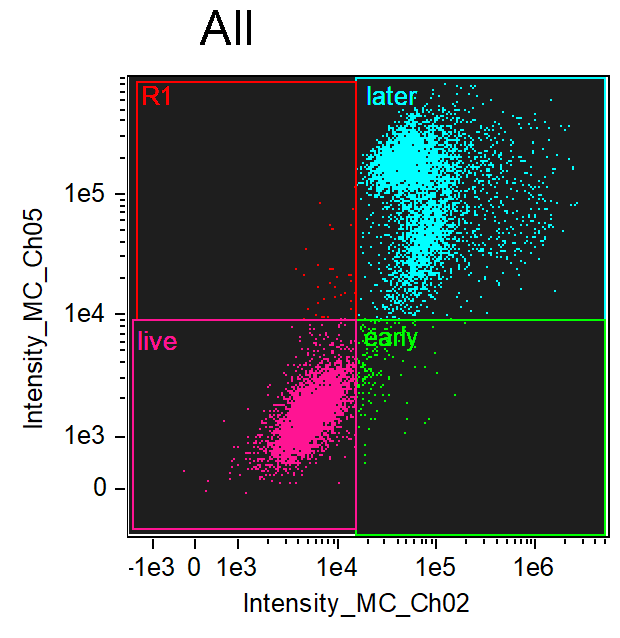


52.1%

1.6%

46%


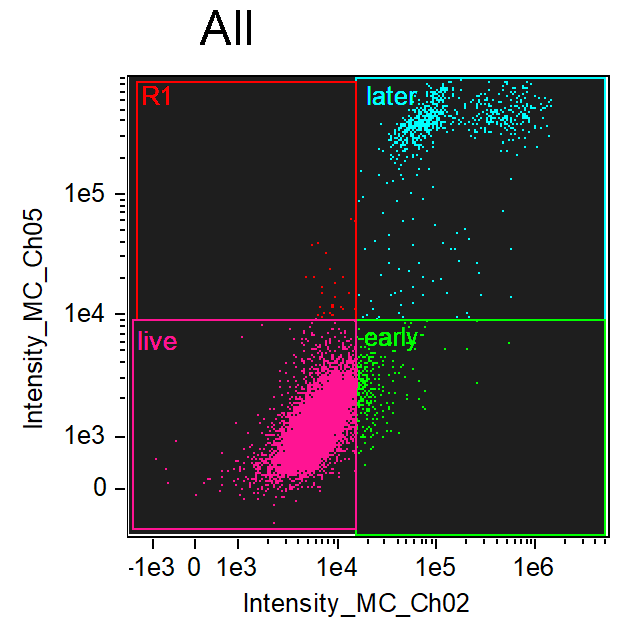


control

7.62%

2.82%

89.2%

CpG


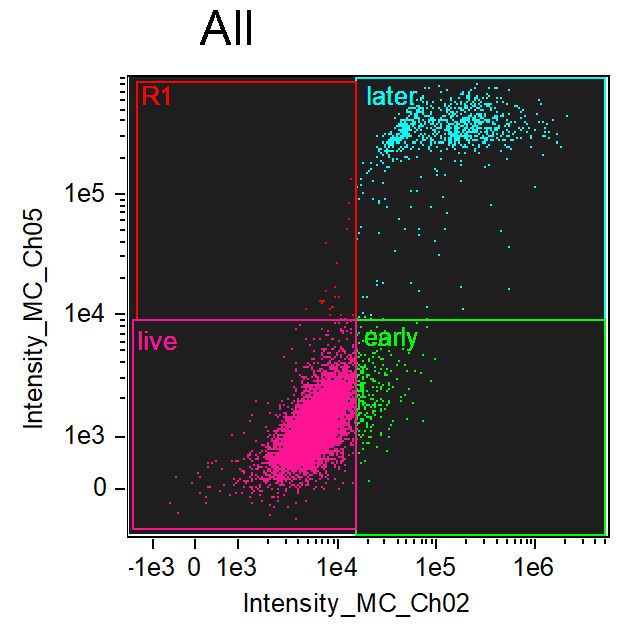


8.86%

2.72%

88.2%

cisplatin


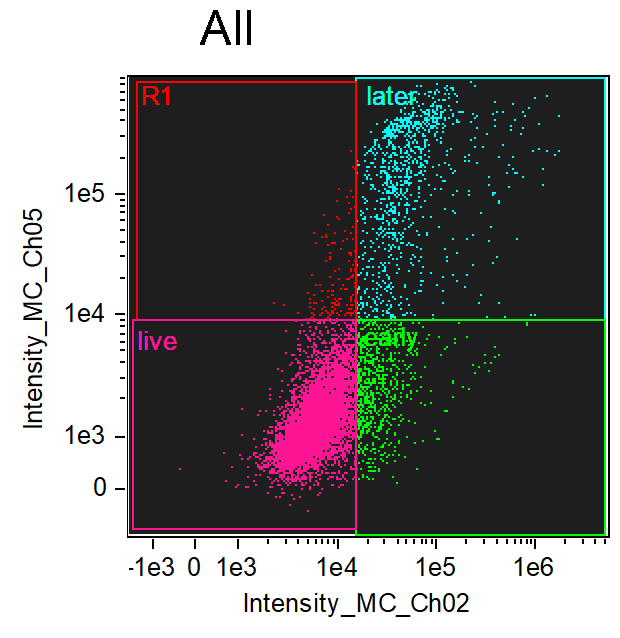


10.7%

7.62%

80.1%

CpG+cisplatin


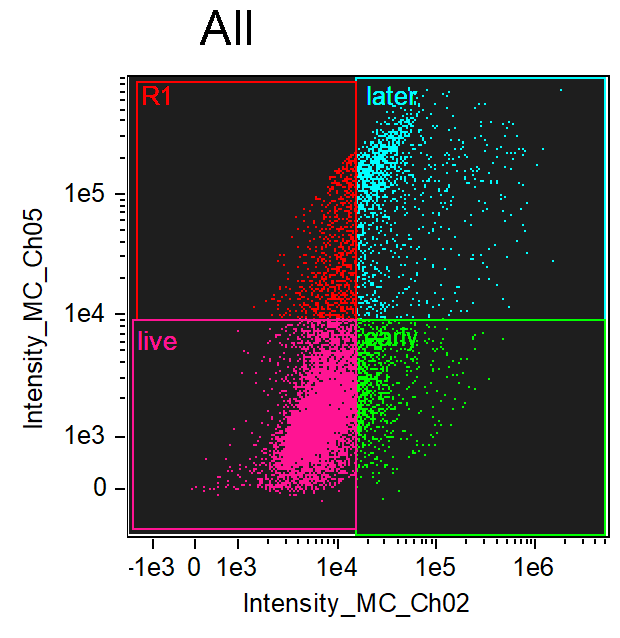


12.8%

8.42%

71.1%

Annexin V-FITC

Propidium Iodide

(b)

0

0.2

0.5

0

50

100

150

**

**

t-BHP（mM）

Cell Viability (%)

(a)

Apoptotic cell (%)

0

20

40

60

**

ns

CpG

t-BHP

cisplatin

+

**–**

**–**

+

**–**

**–**

+

**–**

**–**

+

**–**

**–**

+

+

+

**–**

**–**

**–**

(c)

Supplemental figure 2. The role of CpG ODN in t-BHP-induced apoptosis of AML12 cell line. Cisplatin induced apoptosis were used as positive control. The cell viability of AML12 cell was measured by CCK-8 kit with different concentrations (0, 0.2 mM, 0.5 mM) of t-BHP (Supplemental figure 2(a)). AML-12 cells were pretreated with CpG for 1 hour, after stimulation of t-BHP (500 µM) for 6 hours and cisplatin (40 µg/ml) for 24 hours. The percent of apoptotic cell was measured by Annexin V/PI dye and analyzed by Amnis ImageStream Markǁ Imaging Flow Cytometer workflow (Supplemental figure 2(b) and 2(c)). The scatter plot diagrams of apoptosis are analyzed by IDEAS^TM^ v6.2 and the histogram was required from Graphpad prism 7.0. All results were expressed as mean ± SEM of three independent experiments. ^*^ *P* < 0.05;^**^ *P* <0.01; ns=no significant difference , n = 3.
